# Placebo can enhance creativity

**DOI:** 10.1371/journal.pone.0182466

**Published:** 2017-09-11

**Authors:** Liron Rozenkrantz, Avraham E. Mayo, Tomer Ilan, Yuval Hart, Lior Noy, Uri Alon

**Affiliations:** 1 Theater lab, Weizmann Institute of Science, Rehovot, Israel; 2 Dept. Molecular Cell Biology, Weizmann Institute of Science, Rehovot, Israel; 3 Dept. Neuroscience, Weizmann Institute of Science, Rehovot, Israel; Kyoto University, JAPAN

## Abstract

**Background:**

The placebo effect is usually studied in clinical settings for decreasing negative symptoms such as pain, depression and anxiety. There is interest in exploring the placebo effect also outside the clinic, for enhancing positive aspects of performance or cognition. Several studies indicate that placebo can enhance cognitive abilities including memory, implicit learning and general knowledge. Here, we ask whether placebo can enhance creativity, an important aspect of human cognition.

**Methods:**

Subjects were randomly assigned to a control group who smelled and rated an odorant (n = 45), and a placebo group who were treated identically but were also told that the odorant increases creativity and reduces inhibitions (n = 45). Subjects completed a recently developed automated test for creativity, the creative foraging game (CFG), and a randomly chosen subset (n = 57) also completed two manual standardized creativity tests, the alternate uses test (AUT) and the Torrance test (TTCT). In all three tests, participants were asked to create as many original solutions and were scored for originality, flexibility and fluency.

**Results:**

The placebo group showed higher originality than the control group both in the CFG (p<0.04, effect size = 0.5) and in the AUT (p<0.05, effect size = 0.4), but not in the Torrance test. The placebo group also found more shapes outside of the standard categories found by a set of 100 CFG players in a previous study, a feature termed out-of-the-boxness (p<0.01, effect size = 0.6).

**Conclusions:**

The findings indicate that placebo can enhance the originality aspect of creativity. This strengthens the view that placebo can be used not only to reduce negative clinical symptoms, but also to enhance positive aspects of cognition. Furthermore, we find that the impact of placebo on creativity can be tested by CFG, which can quantify multiple aspects of creative search without need for manual coding. This approach opens the way to explore the behavioral and neural mechanisms by which placebo might amplify creativity.

## Introduction

The potency and mechanism of the placebo effect are increasingly researched. The placebo effect is due to the psychobiological phenomena that occur with the administration of the inert substance[1]. These psychobiological phenomena can be induced by expectation, verbal suggestions and classical conditioning [2–4] [5, 6].

Most studies of placebo so far have been in clinical settings with the goal of decreasing negative symptoms such as pain, depression and anxiety. These studies suggest several neurobiological pathways for placebo, which can be differentially activated in different contexts[7]. Analgesia placebo, the best understood placebo to date, is characterized by activation of endogenous opioids and dopamine to reduce spinal nociceptive responses[8]. This pathway provides evidence of how high-order processes—such as expectation—can regulate immediate peripheral sensations such as pain[9]. Placebo has also been widely studied in the treatment of Parkinson’s disease. Here, placebo involves the dorsal striatum, which plays a role in motor control, and dopamine release in the ventral striatum, which is part of the reward system[10]. Activation of the reward system has also been shown to affect immune states in mice[11].

There have been much fewer studies on using placebo outside of the clinic in order to enhance positive aspects of performance or cognition. Several studies showed that placebo can improve sports performance (reviewed in [9, 12]). Most of these studies were on professional athletes and used an inert substance or treatment together with suggestions. The athletes were told they were receiving an ergogenic aid (anabolic steroids, caffeine etc.), when in fact they received a placebo. They were then tested for their endurance or strength in the relevant field. Some studies administered an active substance alongside the placebo and some administered only placebo but manipulated expectations regarding its effect. In diverse athletic fields ranging from anaerobic sprint runs and weightlifting to long-range aerobic endurance cycling, placebo extended performance in an expectation-dependent manner. For example, if subjects expected a higher dose of caffeine, they had higher performances, and if they expected negative effects of the substance, performance worsened [13–16]. Pre-conditioning strengthened the placebo effect. For example, subjects received a placebo said to be caffeine, and then were tested for lifting a weight which was reduced without their knowledge. Then they received the same placebo-caffeine, and this time tested on the original weight. Performance was improved relative to a group which received placebo-caffeine in one session only [17, 18].

Several other studies tested the ability of placebo to enhance cognitive performance. In these studies there was no comparison to an active substance. Instead, the independent variable was subjects’ expectations manipulated by means of suggestion: a group told that a sham drug or intervention will improve performance (placebo group) is compared to a group going through the same procedure with no mention of improvement (control group). For example, Parker and colleagues showed that an inert substance presented as a drug that acts as a cognitive enhancer increased performance on a prospective memory task, compared with a group which received the same substance and was told it was an inactive control. Prospective memory improvement was at the expense of response times in an ongoing task performed in parallel, indicating increased cognitive effort[19]. Oken and colleagues showed that an inert pill, which was said to be a cognitive enhancer, improved various cognitive abilities in healthy seniors. The control group were same-age subjects which went through the same procedures but were not given the pills. Regression analysis indicated that expectancy, self-efficacy and perceived stress were significant predictors for placebo-related improvement[20].

Placebo was also found to enhance performance in subconscious cognitive tasks. For example, performance on the Stroop effect, a classical response-time test in cognitive psychology, was improved by a sham EEG[21] or by verbal suggestion[22]. More specifically, in the former study, de Gama et al used a within-subjects design that compared performance on the Stroop task at baseline versus -performance during sham EEG said to modulate participants’ visual ability to perceive colors: either to enhance it or to decrease it. Participants exhibited less or more interference from written color words in accordance with the corresponding suggestion[21]. In another subconscious implicit learning trial, Colagiuri et al told subjects that an odor influences performance, either positively (placebo group 1), negatively (placebo group 2), or not at all (control). Subjects completed the task in alternating blocks in which odor was presented or not presented. The study found that reaction times on cued trials were faster or slower according to the placebo suggestion[23]. Finally, Weger et al. used a sham subliminal priming procedure which was said to unconsciously enhance subjects’ knowledge. Performance on a general knowledge test was enhanced compared to a control group [24]. Based on these encouraging findings, there is scope to explore placebo for improving additional positive aspects of human performance.

Here, we ask whether placebo can enhance creativity. Creativity is the ability to generate ideas, solutions or insights that are new and potentially useful[25–27]. Creativity is often viewed as a trait characteristic of a person; however, creativity can also be viewed as a *state*, affected by expectation and motivation[28–30]. Motivation appears to be a central factor in creative performance[25, 31–33], a finding which is hopeful because motivation can be bolstered, for example, by enhancing belief in one’s own competence[34, 35]. In this regard, Green et al found that a suggestion to be more creative increased novelty scores in a word association task, and several studies indicated that conditions that reduce inhibitions can enhance performance on creativity tests[28, 36–39].

There is much current interest in finding ways to enhance the creativity of individuals and groups [40–42]. One obstacle in the study of creativity is the lack of experimental paradigms that allow automated and multi-dimensional views of the creative process. Current paradigms, such as the alternate uses test (AUT) and the Torrance test of creative thinking (TTCT), require laborious manual coding, and do not allow access to the process or intermediate steps by which solutions are reached. A recent advance presented an automated test for creativity called the creative foraging game (CFG, [43]). The CFG is a computer game in which participants search for interesting and beautiful shapes in a well-defined geometric space. This test allows measurement of multiple aspects of the search including fluency, uniqueness of solutions and the length and timing of the paths taken to reach the solutions.

Here, we hypothesize that a placebo that combines an inert substance with a verbal suggestion aimed at increasing creativity and reducing inhibitions will increase subjects’ creativity. We used an odorant as an inert placebo substance, which is less invasive than pills or injections and hence appropriate for non-clinical settings. We tested creativity with two standard manual tests, AUT and TTCT, and with the automated creative foraging game to compare aspects of the creative search between groups that smelled the odorant with and without the suggestion.

## Methods

### Participants

Participants were recruited between March 2014 and June 2015 via social media groups dedicated to recruiting subjects for experiments, and were mostly students from nearby universities. Compensation was 40 NIS (about 10$) per hour. Ninety-six participants took part in the experiment. Before analysis, we removed three participants who were subject to another suggestive experiment directly before the current one, two participants with abnormally short games and one participant who had previous knowledge of the research hypothesis. Data from ninety participants entered the analysis. All participants were blindly assigned to placebo (N = 45) or control (N = 45) groups prior to their arrival in the laboratory. There were no significant age and sex differences between the two groups (placebo: 49% males, mean age 25.4±2.5, age range 20–33; control: 49% males, mean age 26.1±3.2, age range 21–37). Of these 90 participants, 57 participants (30 from the control group and 27 from the placebo group) completed two additional creativity tests (see study design). Here too, no age and sex differences were found (placebo: 52% males, mean age 25.8±2.6, age range 22–33; control: 50% males, mean age 26.2±3.1, age range 21–32). This study was approved by our Institutional Review Board, Wolfson Medical Center Helsinki Committee. All patients provided written informed consent.

### Study design

The experiment took place in an olfactory research lab, in which experiments typically ask subjects to rate odors and perform tasks. Participants signed informed consent, came into the experiment room, were presented with an odorant in a jar and were asked to smell it, and to rate its pleasantness, familiarity and intensity. Importantly, the placebo group was also told that the odorant is “a unique odor, developed in our lab, which increases creativity and lowers inhibitions”. All aspects of the experiment, except the suggestion, were identical between the two groups; the only thing that differed between their experiences was what we told them about the nature of the substance.

Following the odorant presentation, the participants were introduced to the creative foraging game (CFG), and played it for 10 minutes. Fifty-seven participants (30 from the control group and 27 from the placebo group) continued the experiment as followed: They were asked to smell and rate the odorant again, the placebo group were told this was to maintain the effect of the odor and the control group were told it was to get odor ratings at different time points, and then completed two classical creativity tests—the alternate uses test (AUT) and a subset of Torrance test of creative thinking (TTCT), in a counter-balanced order, each for 10 minutes as well. The total duration of the experiment was approximately 40 minutes.

### The odorant

We used a component of the odor of cinnamon (cinnamaldehyde, Sigma-Aldrich, CAS 104-55-2). Participants rated the odor on a visual analog scale as mildly pleasant (normalized scores to a scale of 1–100: 64.2 ± 22.8), mildly familiar (65.5 ± 25) and relatively intense (73.3 ± 16.4). There were no significant differences between the placebo and control groups in odor ratings (pleasantness: MW U = 950, p = 0.61; two-tailed t_88_ = 0.54, p = 0.59; familiarity: MW U = 989, p = 0.85; two-tailed t_88_ = 0.15, p = 0.89; intensity: MW U = 776, p = 0.06; two-tailed t_88_ = 1.85, p = 0.07, a trend towards the placebo group to perceive the odor as more intense). This result rules out the possibility that differences in group performance were due to differential perception of the odor. There was no significant correlation between odor ratings and performance on the creativity tests (S1 Table). There is evidence that cinnamon odor increases attention and memory[44, 45], although conflicting evidence[46] was also reported. Since both groups were equally exposed to the odor, any presumed effect of the odor itself would affect both groups.

### Creativity tasks

We used three creativity tests: the Creative Foraging Game (CGF[43]) in which participants are asked to search for interesting and beautiful shapes in a defined geometric space, the Alternate Uses Test (AUT[47]) which is a verbal test for divergent thinking, and the figural Torrance test (TTCT[48]) which is a visual test for divergent thinking. Scoring of all tests was blind for condition.

### Creative foraging game (CFG)

**Fig 1:**
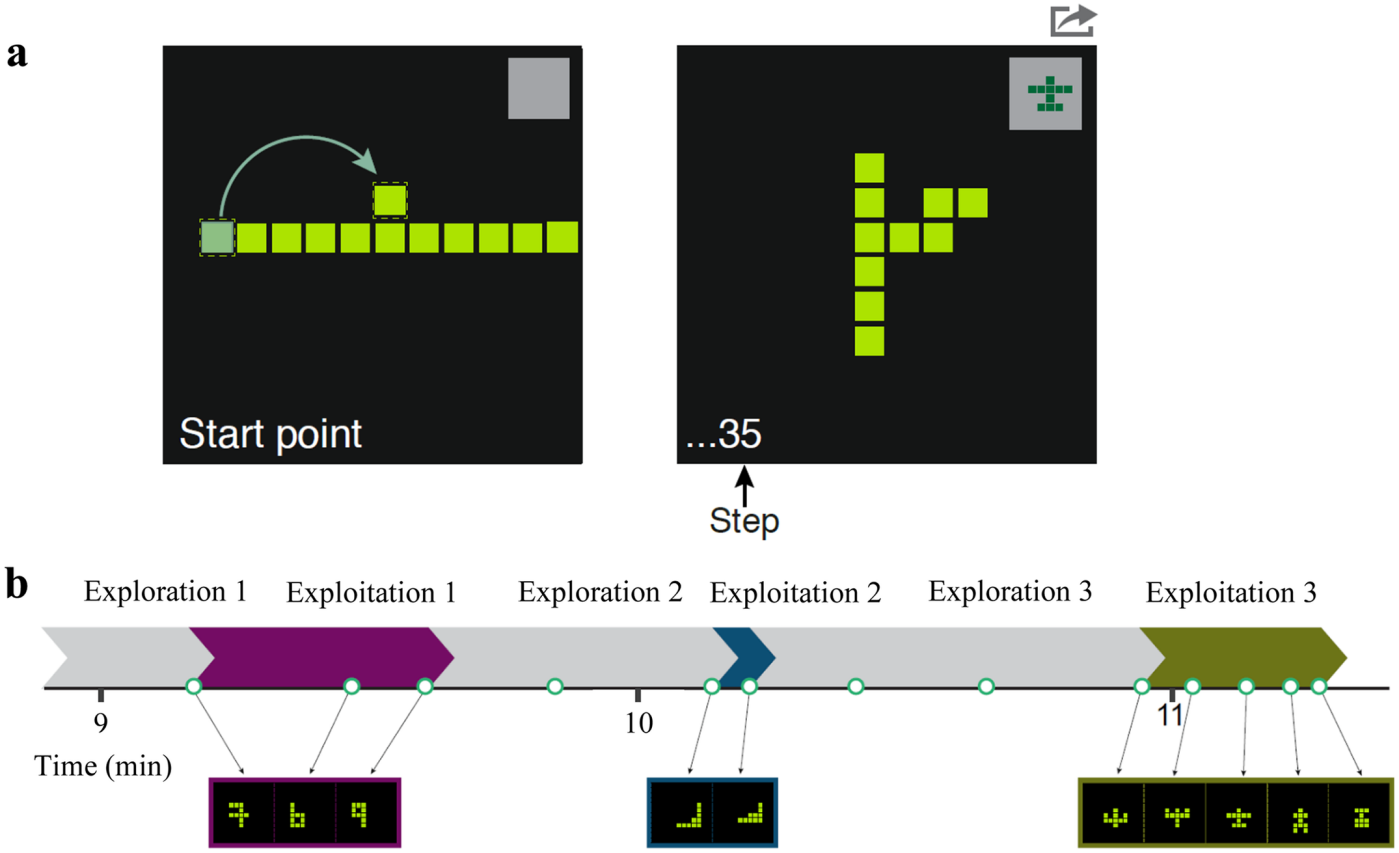
Creative foraging game (CFG). a: CFG interface. Left: starting point; right: example of a shape. b: Exploration phases are followed by exploitation phases in which participants usually find shapes with shared perceptual meaning. Purple: numbers; green: airplanes. Data for fig 1b is from one of the participants from Ref [43].

The creative foraging game (CFG,[43]) is a computer game in which participants create shapes by moving one of ten identical squares at each step, keeping the squares connected by an edge (see Fig 1a). The initial condition is ten squares in a horizontal line. Participants are asked to explore the space of possible shapes (a total of 36,446 shapes), and when they come across a shape they find interesting or creative, to select it to a gallery. This is done by pressing a gray square at the top-right side of the screen, which saves the current shape to the gallery (Fig 1a). The gallery has no limit on the number of shapes. After the game, players perform another task—choosing the five most creative shapes from their gallery. This task is not analyzed in the current study. The automated algorithm allows constant recording and analysis of players’ moves and reaction times.

We use the method described in Hart et al [43] to analyze the CFG. During the game participants alternate between two phases termed *exploration* and *exploitation*[43]. The start and end time points of these phases are automatically defined by a segmentation algorithm. The algorithm uses the time points of choosing gallery shapes as the input. Briefly, exploration phases are defined by increasing time intervals between gallery choices and exploitation phases are defined by decreasing time intervals between gallery choices (see ref [43] for details). Exploration phases can include a single gallery shape.

As shown in Ref [43], gallery shapes found in a given exploitation phase have shared perceptual meaning, and are consistently re-discovered by different participants. By clustering the shapes that are re-discovered by different participants, Hart et al. defined shape categories of meaning (SCM). Three examples of SCMs are shapes that resemble airplanes, numerical digits and letters (Fig 1b). Other SCMs include more abstract shapes with visual similarity. In Ref [43], 14 shape categories of meaning were found and were reliably distinguished in a separate discrimination experiment in which people who did not play the game classified shapes from the same SCM versus shapes from another SCM.

CFG Scoring: In creativity tests such as AUT and TTCT, experimenters have access to the solutions provided by the participants verbally or in drawn form, but not to intermediate steps or solutions. We reasoned that such forwarded solutions correspond, in the CFG, to the gallery shapes found in exploitation phases, and thus we scored exploitation gallery shapes rather than all gallery shapes. Fluency was scored as the total number of gallery shapes found by a player in all exploitation phases. Originality was scored as follows: each shape in the CFG was assigned a probability to be found, based on a database of 100 games by participants who did not take part in the current study[43]. The originality score of a participant is equal to the average of the minus log of the probabilities of all gallery shapes found in that player’s exploitation phases. The flexibility score of a player is the number of different SCMs found by that player based on the SCMs found in a combined dataset of the 100 players of Ref [43] plus the 45 players in the group tested (control or placebo), plus the number of exploitation gallery shapes that did not fall into any of the SCMs. The out-of-the-boxness (OB) score for each participant was the fraction of exploitation gallery shapes that lie outside of the standard set of SCMs defined by the 100 players of Ref [43]. To test the robustness of this result, we used a random subset of 75 out of the 100 players of Ref [43] as a database for the SCM, and found similar results regarding higher OB in the placebo group. It was not possible to use less than 75 players because it is no longer possible to detect SCMs. Note that OB differs from originality because OB considers categories of shapes rather than specific shapes. OB differs from flexibility because it concerns the fraction of gallery shapes outside the SCMs, rather than the number of different SCMs found.

#### Alternate uses test (AUT)

We followed the protocol of Ref [49]. Participants were given a list of five common items (shoe, pin, sheet, nail and button) and asked to list as many alternate uses as possible for each object within 10 minutes, while trying to think of original uses (the most common everyday use was indicated in parenthesis). Only responses that did not reiterate the given common uses were counted and included. Suggested uses which were meaningless were discarded. Scoring included fluency, originality and flexibility.

AUT scoring: We followed the procedure of Ref [49]. Fluency was scored by the number of alternate uses found for each object averaged over the five objects. Originality was defined as statistically infrequent responses of all responses provided per object. Specifically, for each object, a list of all obtained uses was collected from all participants. Two raters grouped similar uses into groups, with inter-rated agreement of 89.5% (Kappa coefficient 0.79). For example button as an earring/ as jewelry were grouped together, as were shoe to throw at someone/ as a weapon/ to hit someone. An infrequency score was assigned to each group as follows: answers which were listed by 5% or more of the participants were given a score of 0; answers provided by 2–5% were scored 1, and answers less frequent than 2% were scored 2. The total originality score of a participant is the mean over the infrequency score of all responses. Flexibility was scored by the number of different categories (groups) of the solutions of each object. The total flexibility score is the mean over the number of categories for each object of that participant.

#### Torrance test of creative thinking (TTCT), figural part, circles subset

We followed the protocol of the TTCT manual[50]. Participants were given a printed page with 35 identical circles with 1.27 cm (0.5 inch) radii in a 5x7 array. They were asked to draw as many different drawings/ideas as possible within 10 minutes, while trying to make each drawing/idea original and creative. Each drawing must include at least one circle and must be given a title. As before, scoring included originality, flexibility and fluency. Fluency was scored by the number of drawings generated from the circles. Originality and flexibility were both scored based on the TTCT scoring guide[50], which specifies originality scores (0, 1, 2 or 3) for about 150 potential drawings, in about 60 different categories. According to the guidelines, a drawing which is not specified in the manual gets 3 points for originality. As before, the total originality score of a participant is the mean over the originality score of all his/her drawings. When two or more circles are combined to create a single drawing, they are given a bonus originality score, which is added, according to the guidelines, to the total originality score. Flexibility was scored by the number of different categories of the drawings of each participant, using the manual. A drawing which was not mentioned in the manual was given a category according to those specified in the manual, or provided with a novel one, if none would fit. The total flexibility score is the mean over the number of categories of that participant.

### Statistical analysis

To compare the control and placebo groups, we used the Mann-Whitney (MW) non-parametric test, which is appropriate because the data is not necessarily normally distributed. We note here also results for the historically more widely used two-tailed t-test: In all cases where we compared groups of total size N = 90, the t-test gave results almost identical to the MW test: when MW was significant p<0.05 so was the t-test, and when MW>0.05 so was the t-test. In cases where N = 57 (comparison with the AUT and TTCT tests), T-test results sometimes showed a trend p = ~0.1 when the MW test showed significance or trend for significance p<0.06. These cases are AUT originality: MW p = 0.048, T-test p = 0.11; AUT fluency MW p = 0.056, T-test p = 0.12; AUT flexibility MW p = 0.06, T-test p = 0.11. All statistically significant results reported here employed the Benjamini-Hochberg multiple-hypothesis correction using FDR = 0.15.

## Results

The comparison between the placebo and control group is shown in Table 1. We find no significant effect of placebo on the TTCT test, and hereafter we focus on the CFG and AUT tests.

**Table 1:** Performance of the placebo and control groups in the three creativity tests.

Test	Measure	Placebo median ±MAD	Control median ±MAD	p-value MW	Effect size
**Creative Foraging Game (CFG)**	Originality	7.0±0.5	6.7±0.4	0.036*	0.46
	Fluency	14±6.6	16±8.6	0.73	--
	Flexibility	10±7.2	11±6.5	0.96	--
	Out-of-the-boxness	0.47±0.17	0.35±0.15	0.008**	0.6
	Exploitation phases	5±2	5±2.2	0.7	--
**Alternate Uses Test (AUT)**	Originality	3.6±1.3	2.6±1.5	0.048*	0.43
	Fluency	4.2±0.9	3.2±1.1	0.056^†^	0.43
	Flexibility	3.8±0.8	3±1.1	0.06^†^	0.44
**Torrance Test of Creative Thinking (TTCT)**	Originality	28±13	28±9	0.75	--
	Fluency	10±5	8.5±4	0.34	--
	Flexibility	7±3	7±3	0.78	--

** p value < 0.01;

* p value < 0.05;

^†^ trend, p value < = 0.06

### The placebo group showed higher originality

**Fig 2:**
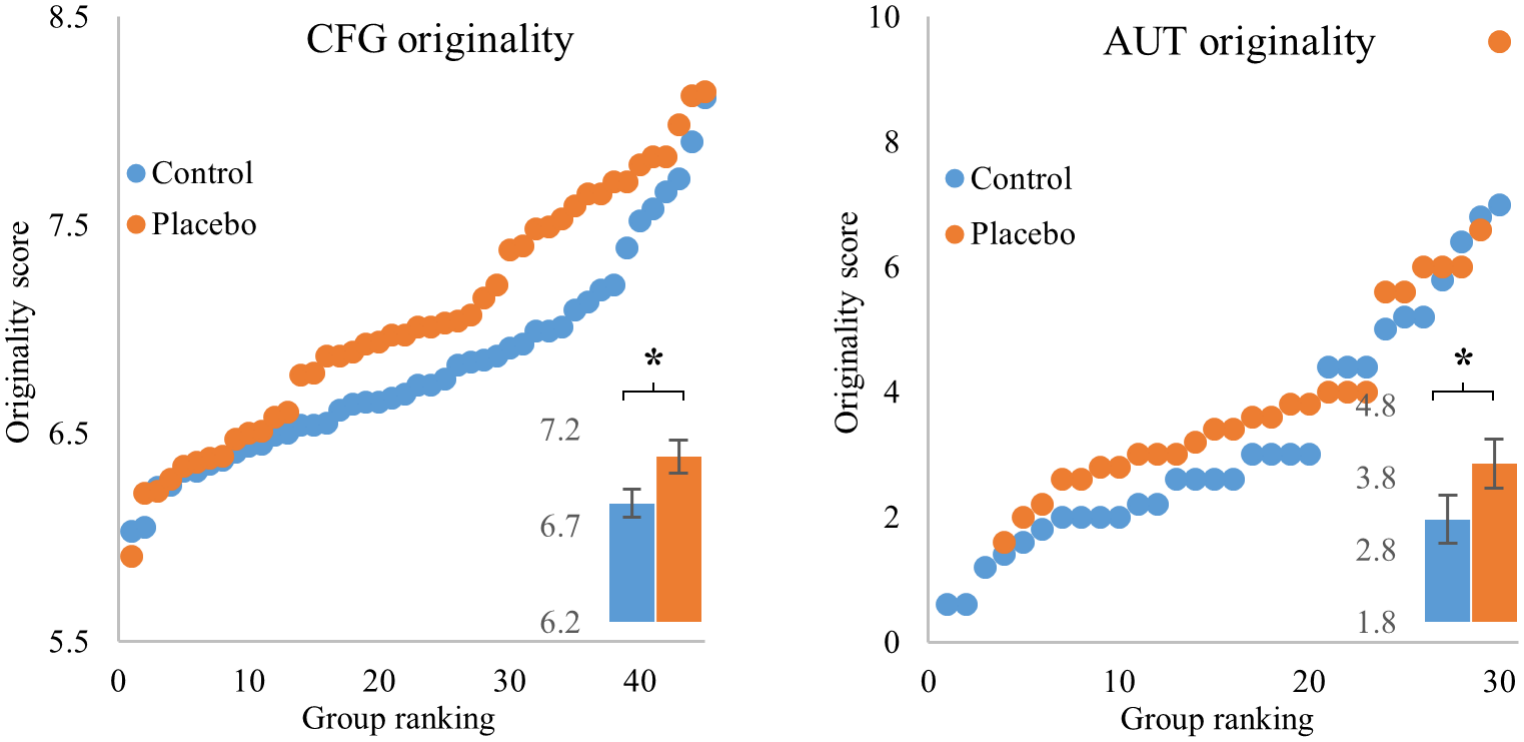
The placebo group showed significantly higher originality in both the CFG (left) and AUT (right). Scatterplots of originality scores of the placebo (orange) and control (blue) groups. Y axis is originality scores, ranked from lowest to highest. X axis is group ranking (in the AUT since number of participants in the placebo group was smaller by 3, matching is from the highest score onwards). Insets show mean originality in each group, error bars are standard error of the mean. * p value < 0.05.

We first consider originality—the extent to which a player finds solutions not found by other participants (Methods). Participants in the placebo group showed significantly higher originality than the control group, both in the CFG (MW U = 753, p = 0.036; Fig 2, left panel), and in the AUT (MW U = 278, p = 0.048, Fig 2, right panel). Effect size was medium (CFG: Cohen’s D = 0.46; AUT: Cohen’s D = 0.43).

### Fluency and flexibility did not significantly differ between the groups in the CFG, and were marginally significant in the AUT, in favor of the placebo group

**Fig 3:**
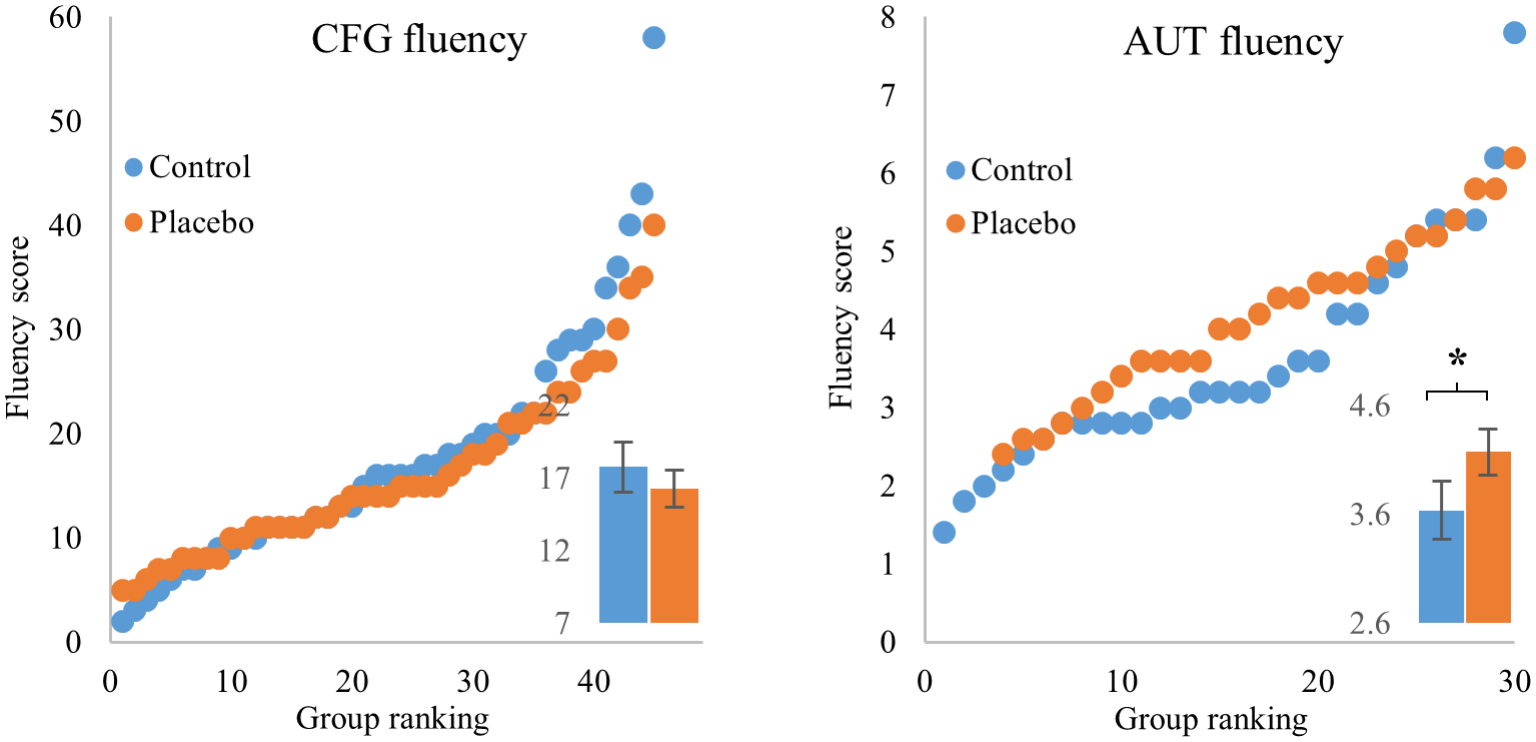
The placebo group showed a trend for higher fluency in the AUT (right) and no such difference in the CFG (left). Scatterplots of fluency scores of the placebo (orange) and control (blue) groups. Y axis is fluency scores, ranked from lowest to highest. X axis is group ranking (in the AUT since number of participants in the placebo group was smaller by 3, matching is from the highest score onwards). Insets show mean fluency in each group, error bars are standard error of the mean. † p value < 0.06.

We next compared fluency, defined as the overall number of solutions. In the CFG, we defined fluency as the number of shapes selected to the gallery during exploitation phases, finding no significant difference between control and placebo (MW U = 970, p = 0.73). In the AUT test, fluency showed a trend for being higher in the placebo group than in the control group (MW U = 286, p = 0.056, Cohen’s D = 0.43) (Fig 3).

We also compared flexibility between the placebo and control groups, defined as the number of categories of suggested solutions. We find that flexibility in the CFG was not statistically different between the placebo and control groups (MW U = 1005, p = 0.96). In the AUT, the placebo group showed a trend towards higher flexibility (MW U = 287, p = 0.06).

### The placebo group showed greater out-of-the-boxness in the CFG

**Fig 4:**
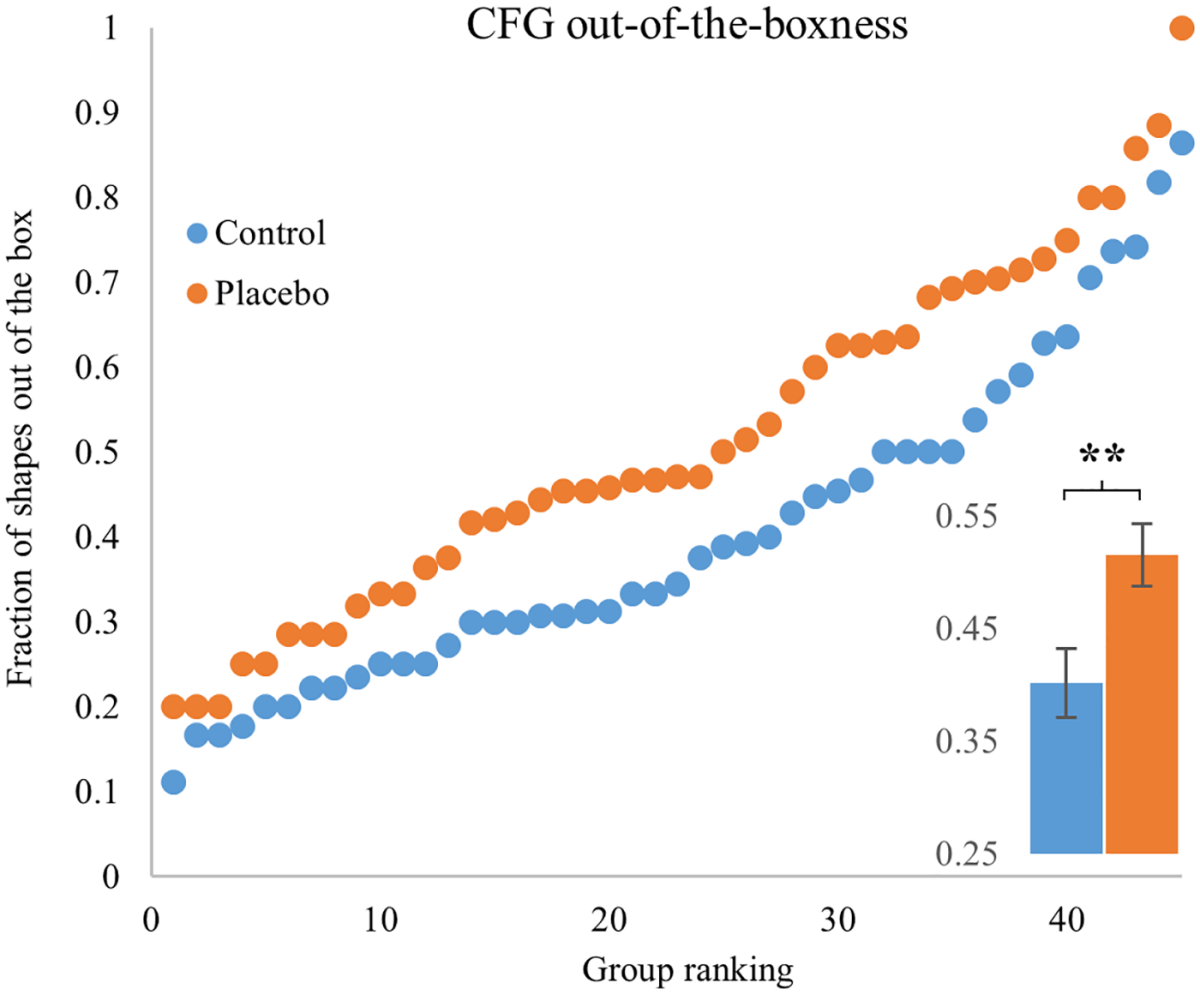
The placebo group displayed higher “out-of-the-boxness” (OB) than the control group in the CFG. Main plot is a scatterplot of OB scores of the placebo (orange) and control (blue) groups. Y axis is the fraction of shapes outside standard categories, ranked from lowest to highest. X axis is group ranking. Inset shows mean OB in each group, error bars are standard error of the mean. ** p value < 0.01.

When comparing flexibility of the two groups in the CFG, we noticed that the placebo group found many shapes that did not fit any category previously discovered by a database of 100 games[43]. We devised a score for this effect, the extent to which players found categories of shapes that were non-standard, naming it “out-of-the-boxness”, OB. We asked, for each participant in our study, what fraction of the gallery shapes lies outside of the standard set of shape categories (SCM, see Methods). Note that OB differs from originality because it considers categories of shapes rather than specific shapes. We find that the OB of the placebo group was significantly higher than the control with medium-to-large effect size (MW U = 683, p = 0.008; D = 0.6). Fig 4 shows that this effect seems to be spread among all 90 players.

## Discussion

We find that placebo can enhance the originality aspect of creativity. We used an odorant together with a verbal suggestion that it enhances creativity and reduces inhibitions. We evaluated creativity using three tests, the classical AUT and TTCT tests and the CFG, an automated creativity test in a well-defined search space. The placebo group showed increased originality in the AUT and CFG, but not the TTCT, by finding solutions rarely found by other players. The placebo group also showed higher out-of-the-boxness in the CFG by finding solutions not included in categories found by a database of 100 players from a previous study. The size of the significant effects was medium to strong, and effects were distributed among most members of the placebo group. Placebo did not seem to strongly affect fluency, the number of solutions found, which suggests that subjects were not simply less selective, but rather genuinely more original.

What are the psychological mechanisms that allow placebo to increase the originality aspect of creativity? There are at least two possibilities. The first mechanism is based on extensive research by Amabile and Deci and Ryan[25, 33, 34, 51–53], that suggests that creativity is modulated by motivation. Extrinsic motivators were shown to be mostly detrimental to creativity, whereas intrinsic motivation is conductive to and strongly associated with creative abilities[25, 32, 33, 51, 54–56]. A key factor in intrinsic motivation, according to self-determination theory [34, 35], is the belief in one’s competence. For example subjects who practiced encouraging statements (related to self-confidence, releasing anxieties etc.) and omitted self-incapacitating statements showed improved creativity scores[57]. This is in line with the verbal suggestion in our study that the odorant increases creativity, which may have made subjects feel more competent. Additional components of intrinsic motivation, such as social relatedness, may also have been increased by experimenter effects in the present study, by the experimenter’s perceived interest in the effects of the odorant.

A second possible psychological mechanism of placebo, as suggested by Weger et al., is to weaken inhibitory mechanisms that normally impair performance[24]. Creativity was found to increase in several studies that tested conditions with reduced inhibitions, such as alcohol consumption[36–38]. Wieth and Zacks showed that creative problem solving was improved when participants were tested during non-optimal times of day, and suggested that this is due to reduced inhibitory control[39]. Moreover, studies which used non-invasive brain stimulation by means of transcranial direct current stimulation (tDCS) found enhanced creativity, and attributed it to reduced inhibitions and diminished cognitive control[49, 58]. This effect was suggested to be in line with paradoxical functional facilitation theory, which attributes improved performance of damaged nervous system to release from inhibition[59]. Informal notions in improvisation theatre suggest that the inner critic is a source of inhibition that limits creativity[60]. The verbal suggestion made in our study that the odorant increases creativity and reduces inhibitions may thus work through a reduced-inhibition mechanism and/or by increasing belief in one’s competence. Future work can test which of these mechanisms is at play.

Our use of three tests for creativity allows a comparison between the different aspects of creativity within and across tests (S2 table). The two manual tests, AUT and TCTT, did not show significant correlation with each other across participants in fluency, flexibility or originality (all spearman coefficients R < 0.17, all p > 0.22). A similar lack of correlation between different manual tests for divergent thinking was reported in previous studies[61–65]. This suggests that each test might measure different aspects of creativity. Encouragingly, we find that the AUT and CFG showed similar results in terms of increased originality by the placebo group, with a smaller effect on fluency. Whereas fluency and originality did not show significant correlation across tests, when correlating the tests using a combined Z-score for fluency and originality, a moderate and significant correlation is found between CFG and AUT (R = 0.27, p = 0.04,[43]). This suggests that the CFG may complement the AUT as a useful test for creativity, with the advantage that CFG is an automated analysis that does not require manual scoring.

It is interesting to note in this regard that in terms of the creative product, the CFG and TTCT are both figural tests, whereas the AUT is verbal. Yet, here the placebo effect was beneficial for the CFG and AUT, but not TTCT. Whereas the reason to this difference is not clear to us, enhanced creativity in AUT but not TTCT was also found in a study on the effect of transcranial direct current stimulation (tDCS) on creativity [49]. The authors attributed the effect to release from inhibition. Interestingly, similar to our results, this study also found enhancement in the originality aspect of creativity, but not in fluency or flexibility. This may further imply that the placebo subjects in the present study were similarly less inhibited.

Limitations of the present study include a between-subjects design which cannot measure the ability of placebo to increase creativity within an individual. A benefit of studying a placebo effect on healthy individuals is that unlike the clinic, it is possible to employ both conditions on the same person to control for intra-subject variability. In this study we decided not to use this option because performing the CFG twice may create a learning curve or a habituation effect we wanted to avoid. In addition, although the order of the AUT and TTCT was counterbalanced, the CFG was always completed first. This ordering effect might have contributed to the significant findings on the CFG, given that participants completed it first and could have been more alert at the beginning of the experiment. The approaching significant findings on the AUT could be due to insufficient power. Future work can use the finding that AUT and CGF both pick up on the effects of placebo, and employ CFG and AUT in a counterbalanced way before and after placebo in order to address this issue. The study is limited to a single culture and context, and future work can explore placebo on creativity in other cultures and contexts.

Further research can explore the mechanisms by which placebo can enhance cognitive and creative abilities. Such exploration can include, in addition to tests to elucidate psychological mechanisms, also physiological and neurological measures of systematic and autonomous changes[66, 67]. Placebo for enhancing cognitive abilities such as creativity may thus be a research field with beneficial potential.

## Supporting information

S1 Dataset Dataset of all subjects and their creativity scores in the three creativity tests. (XLSX)

S1 Table Correlations of performance on creativity tests with odor ratings. Values in the upper table are Spearman correlation coefficients, values in the bottom table are the corresponding p values (not corrected).
(XLSX)

S2 Table Correlations within and across the three creativity tests. Values above the diagonal represent Spearman correlation coefficients, values below the diagonal represent corresponding p values (not corrected).
(XLSX)
